# *Nardostachys jatamansi* inhibits severe acute pancreatitis via mitogen-activated protein kinases

**DOI:** 10.3892/etm.2012.612

**Published:** 2012-06-18

**Authors:** GI-SANG BAE, KYOUNG-CHEL PARK, BON SOON KOO, IL-JOO JO, SUN BOK CHOI, HO-JOON SONG, SUNG-JOO PARK

**Affiliations:** 1 Hanbang Body-Fluid Research Center;; 2 Department of Herbology, School of Oriental Medicine, Wonkwang University, Iksan, Jeonbuk 540-749, Republic of Korea

**Keywords:** *Nardostachys jatamansi*, choline-deficient and ethionine-supplemented diet, severe acute pancreatitis, mitogen-activated protein kinases

## Abstract

Previously, we reported that *Nardostachys jatamansi* (NJ) attenuated cerulein-induced mild acute pancreatitis (AP). In the present study, we investigated the ability of NJ to ameliorate severe acute pancreatitis (SAP) induced by a choline-deficient diet supplemented with ethionine (CDE). An NJ extract was orally administered *ad libitum* via the water during administration of the CDE. After three days, the CDE was replaced with a normal diet. After four days of normal feeding the mice were sacrificed and the blood and pancreas were obtained for further investigation. NJ treatment reduced SAP-induced pancreatic damage, as shown by histology. NJ treatment also inhibited neutrophil infiltration into the pancreas. NJ also inhibited the secretion of digestive enzymes and cytokine production, and inhibited the activation of mitogen-activated protein kinases (MAPKs) in the SAP-challenged pancreas. These data suggest that NJ protects against pancreatic injury in CDE-induced SAP by deactivating MAPKs.

## Introduction

The mortality and morbidity of acute pancreatitis (AP) generally depend on the severity of the disease, however, the mechanisms regulating its severity are poorly understood. Numerous studies have investigated the processes that regulate the severity of AP using murine AP models (1). Mild edematous AP may resolve either spontaneously or after conservative therapy, but severe hemorrhagic pancreatitis may cause multiple organ failure, leading to a high mortality rate (2). The pathophysiology underlying severe acute pancreatitis (SAP) is not well-understood.

*Nardostachys jatamansi* (NJ) is widely used as a bitter tonic and anti-spasmodic (3). The NJ root contains various sesquiterpenes, including jatamansic acid, and jatamansones, lignans and neolignans. The aqueous extract of the NJ root has been used to treat mental disorders, insomnia and blood disorders (3). We previously reported that NJ is effective in protecting against inflammatory challenges (4–6), particularly against cerulein-induced edematous mild AP (4). At relatively low doses (in line with doses of molecular inhibitors), NJ protected against, and induced recovery from, mild edematous AP. NJ also reduced cytokines, neutrophil infiltration and digestive enzymes. However, the effects of NJ on CDE-induced hemorrhagic severe necrotic AP have not been examined.

The present study was designed to investigate the effects of NJ on CDE-induced SAP. To achieve this, we examined histological changes in the pancreas as well as neutrophil infiltration, digestive enzyme production and cytokine release. We also measured the regulating mechanisms, including mitogen-activated protein kinases (MAPKs).

## Materials and methods

### Materials

Avidin-peroxidase, Tris-HCl, NaCl, hexadecyltrimethylammonium bromide, ethionine and tetramethylbenzidine were purchased from Sigma-Aldrich (St. Louis, MO, USA). Anti-mouse tumor necrosis factor (TNF)-α, interleukin (IL)-1β and IL-6 antibodies and recombinant TNF-α, IL-1β and IL-6 were purchased from R&D Systems (Minneapolis, MN, USA). Anti-phospho-extracellular signal-regulated kinases (ERK) 1/2, anti-phospho-c-Jun N-terminal kinases (JNK) and anti-phospho-p38 were purchased from Cell Signaling Technology (Beverly, MA, USA). Anti-inhibitory κ-Bα (Iκ-Bα), ERK 1/2, JNK, p38 and β-actin antibodies were purchased from Santa Cruz Biotechnology Inc. (Santa Cruz, CA, USA).

### Plant materials

NJ was purchased from a standard commercial source (Omni Herb, Seoul, Korea). The herb identity was confirmed at the Korean drug test laboratory. The NJ was prepared by decocting 100 g of dried herb with 1 l of boiling distilled water for approximately 2 h. The aqueous extract was frozen at −80°C then freeze-dried to form a 6.59-g powder. The yield of the extract was 6.59%. The powder was then rehydrated with distilled water, filtered and the filtrates were stored at 4°C until use.

### Animals

All experiments were performed according to methods approved by the Animal Care Committee of Wonkwang University. Female C57BL/6 mice (aged 3–4 weeks) were purchased from Orient Bio (Sungnam, KyungKiDo, Republic of Korea). All animals were bred and housed in standard shoebox cages in a climate-controlled room with an ambient temperature of 23±2°C and a 12-h light-dark cycle. The animals were fed a standard laboratory diet and water *ad libitum* for seven days prior to random assignment to experimental groups. The mice were fed the choline/methionine-deficient diet (Harland Teklad Madison, WI, USA) supplemented with 0.5% DL-ethionine for three days. To ensure equal exposure by all animals, the diet was replaced with fresh CDE every 24 h. Following CDE administration, animals were provided with a normal diet for four days in order to estimate the 7-day mortality rate. The mice were then sacrificed, and the blood and pancreas were obtained. The blood samples were used to determine serum amylase, lipase and cytokine levels. For histological examination and scoring, the entire pancreas was rapidly removed from each mouse and fixed in formalin. Three portions of each pancreas were stored at −80°C for later measurement of tissue myeloperoxidase (MPO) activity as an indicator of neutrophil sequestration and for real-time reverse-transcription polymerase chain reaction (RT-PCR) measurements. NJ was dissolved in saline, then administrated orally *ad libitum* via water for seven days. There was no significant difference in the consumption of saline and NJ-containing saline.

### Measurement of amylase and lipase

The arterial blood samples were obtained 6 h after induction of pancreatitis for the measurement of serum amylase and lipase levels. The mice were anesthetized with an intraperitoneal injection of ketamine (80 mg/kg) and xylazine (4 mg/kg). Following anesthetization, blood was aspirated from the heart into a syringe. Serum amylase was measured using ADIVA 1650 (Bayer, Leverkusen, Germany). Serum lipase level was measured using a Cobas Mira apparatus (Roche, Basel, Switzerland).

### Enzyme-linked immunosorbent assay (ELISA)

An ELISA for TNF-α, IL-1β and IL-6 (R&D Systems) was carried out in duplicate in 96-well plates (Nunc, Denmark) coated with 100 µl of anti-mouse TNF-α, IL-1β or IL-6 monoclonal antibodies (1.0 µg/ml) in phosphate-buffered saline (PBS) at pH 7.4 following an overnight incubation at 4°C. The plates were washed in PBS containing 0.05% Tween-20 and blocked with PBS containing 10% FBS for 2 h. After additional washes, the standards and serum were added to the plates and incubated at room temperature for 3 h. The wells were then washed and 0.2 µg/ml of biotinylated anti-mouse TNF-α, IL-1β or IL-6 was added to each well and incubated at room temperature for 1 h. The wells were washed, avidinperoxidase was added and the plates were incubated for 30 min at room temperature. The wells were washed again and 3,3′,5,5′-tetramethylbenzidine substrate was added. Color development was measured at 450 nm using an automated microplate ELISA reader. Standard curves were obtained for each sample by using serial dilutions of recombinant TNF-α, IL-1β and IL-6.

### Messenger RNA (mRNA) expression

The mRNA transcripts were analyzed by RT-PCR in mouse pancreatic tissue. The total RNA was isolated from the mouse pancreata using TRIzol (Invitrogen, Carlsbad, CA, USA) and subjected to reverse transcription using SuperScript II RT (Invitrogen). TaqMan quantitative RT-PCR was performed using a LightCycler 2.0 system according to the manufacturer’s instructions (Roche, Basel, Switzerland). Each reaction was performed in triplicate and a control reaction (without reverse transcription) was performed. All samples were analyzed for expression of the gene of interest and the results were normalized to those of the housekeeping mRNA, hypoxanthine-guanine phosphoribosyltransferase (HPRT). Arbitrary expression units were calculated by dividing the expression of the gene of interest by that of *HPRT*. The forward, reverse and probe oligonucleotide primers for Multiplex Real-Time TaqMan PCR were as follows: mouse TNF-α (forward, 5′-TCTCTTCAAGGGACAAGGCTG-3′; reverse, 5′-ATAGCAAATCGGCTGACGGT-3′; probe, 5′-CCCGACTACGTGCTCCTCACCCA-3′), mouse IL-1β [forward, 5′-TTGACGGACCCCAAAAGAT-3′; reverse, 5′-GAAGCTGGATGCTCTCATCTG-3′; universal probe, M15131.1 (Roche Applied Science)], mouse IL-6 [forward, 5′-TTCATTCTCTTTGCTCTTGAATTAGA-3′; reverse, 5′-GTCTGACCTTTAGCTTCAAATCCT-3′; universal probe, M20572.1 (Roche Applied Science)].

### Estimation of MPO activity

Neutrophil sequestration in the pancreas was quantified by measuring tissue MPO activity. The tissue samples were thawed, homogenized in 20 mM phosphate buffer (pH 7.4) and centrifuged (15,000 rpm, 10 min, 4°C). The pellet was resuspended in 50 mM phosphate buffer (pH 6.0) containing 0.5% hexadecyltrimethylammonium bromide. The suspension was subjected to four cycles of freezing and thawing and was further disrupted by sonication for 40 sec. The sample was then centrifuged (15,000 rpm, 5 min, 4°C) and the supernatant was used for the MPO assay. The reaction mixture consisted of the supernatant, 1.6 mM tetramethylbenzidine, 80 mM sodium phosphate buffer (pH 5.4) and 0.3 mM hydrogen peroxide. This mixture was incubated at 37°C for 110 sec, the reaction was terminated with 2 mol/l H_2_SO_4_ and the absorbance was measured at 450 nm.

### Western blotting

The pancreatic tissues were retrieved from storage at −80°C and homogenized in RIPA lysis buffer. Whole-cell lysates were obtained by boiling the homogenates in sample buffer [62.5 mM Tris-HCl, pH 6.8, 2% sodium dodecyl sulfate (SDS), 20% glycerol and 10% 2-mercaptoethanol]. Lysate proteins were then separated by 10% SDS-polyacrylamide gel electrophoresis and transferred to PVDF membranes. The membrane was blocked with 5% skimmed milk in PBS-Tween-20 for 1 h at room temperature and then incubated with anti-phospho-ERK 1/2, anti-phospho-JNK, anti-phospho-p38 and anti-Iκ-Bα antibodies. After four washes in PBS-Tween-20, the blot was incubated with the secondary antibody for 1 h. Antibody-specific proteins were visualized by an enhanced chemiluminesence detection system according to the manufacturer’s instructions (Amersham Corp.).

### Statistical analysis

The results are expressed as the mean ± SEM of independent experiments. Independent one-way ANOVAs were used to analyze the statistical significance of the results between or among groups. All statistical analyses were performed using SPSS version 10.0 statistical analysis software (SPSS, Chicago, IL, USA). P<0.05 was considered to indicate a statistically significant result.

## Results

### NJ attenuated the severity of CDE-induced pancreatic damage

**Figure 1:**
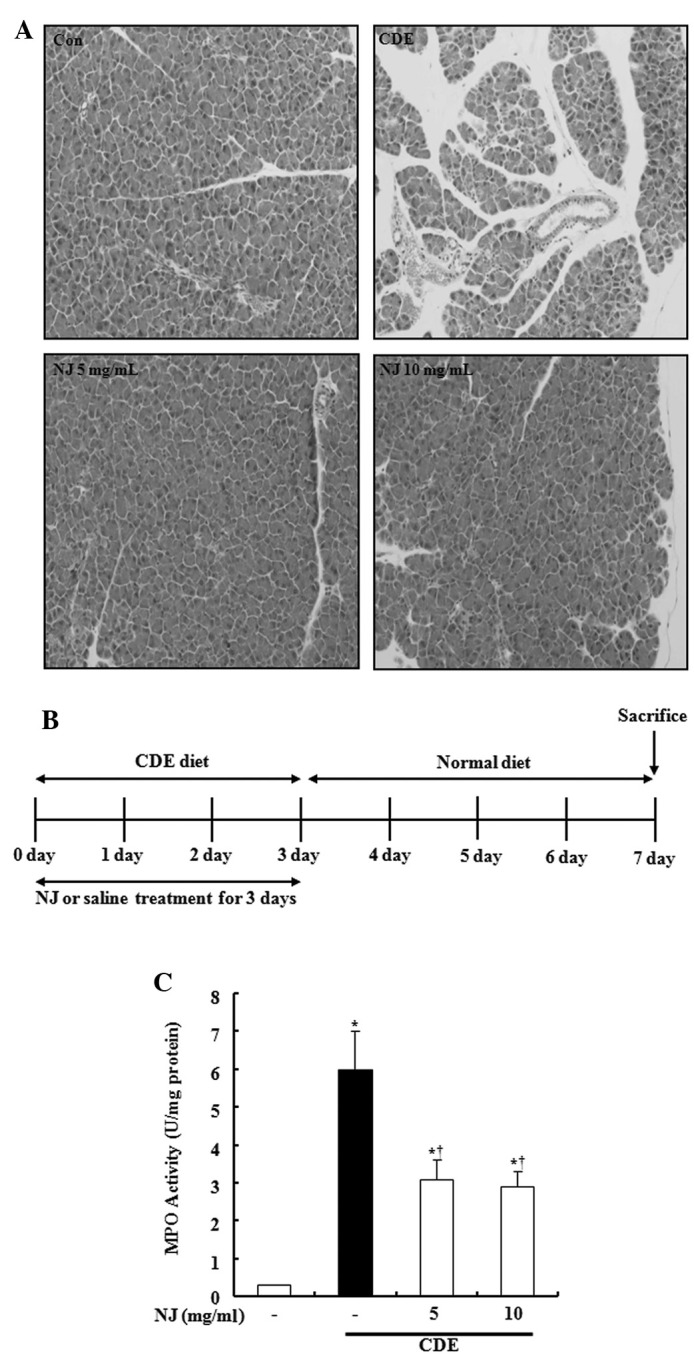
Effect of NJ on CDE-induced SAP. (A) Magnification (×100) of representative hematoxylin and eosin (H&E)-stained pancreas sections of control mice and mice pre-treated with NJ (5 or 10 mg/ml). (B) The treatment method. (C) MPO activity was measured in the pancreas. Data are represented as the means ± SEM (n=6 in each group). ^*^P<0.05 vs. control group and ^†^P<0.05 vs. CDE-induced SAP mice. P<0.05 was considered to indicate a statistically significant result. The results were similar in 3 additional experiments. NJ, *Nardostachys jatamansi*; CDE, choline-deficient diet supplemented with ethionine; SAP, severe acute pancreatitis; MPO, myeloperoxidase.

To examine the effect of NJ on the development and severity of AP, mice were co-treated with NJ (5 or 10 mg/ml) and CDE-induced SAP. The severity of CDE-induced pancreatitis was assessed by histological examination. Pancreatic sections obtained seven days after the onset of SAP (CDE for three days and normal diet for four days, Fig. 1B) revealed the extent of the tissue injury. There was inflammatory cell infiltration of the pancreas and interstitial edema in SAP mice. However, treatment with NJ (5 or 10 mg/ml) attenuated the severity of pancreatitis (Fig. 1A). We also investigated the amount of neutrophil infiltration into the pancreas by assessing the MPO activity. As hypothesized, pre-treatment with NJ significantly inhibited the MPO activity (Fig. 1C).

### NJ significantly inhibited secretion of serum amylase and lipase

**Figure 2:**
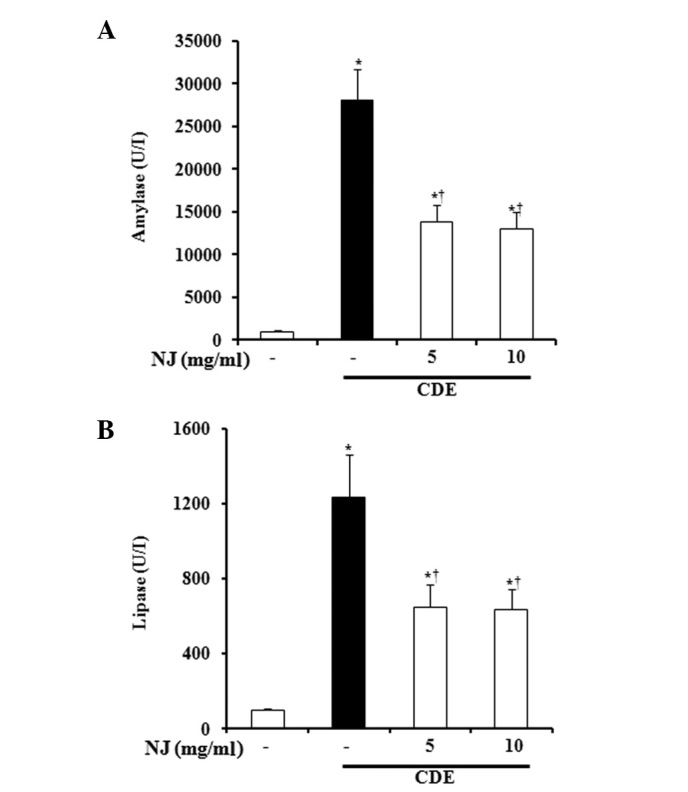
Effect of NJ on the production of digestive enzymes. Serum samples were used to measure (A) amylase and (B) lipase. Data are represented as the means ± SEM (n=6 in each group). ^*^P<0.05 vs. control group and ^†^P<0.05 vs. CDE-induced SAP mice. P<0.05 was considered to indicate a statistically significant result. The results were similar in 3 additional experiments. NJ, *Nardostachus jatamansi*; CDE, choline-deficient diet supplemented with ethionine; SAP, severe acute pancreatitis.

During AP, the digestive pro-enzymes are converted into their active forms, leading to acinar cell death. Therefore, amylase and lipase secretion into the serum signals the initiation of AP. We assessed SAP severity by measuring enzyme production. During CDE-induced SAP, amylase and lipase levels in the serum were significantly increased. However, NJ (5 or 10 mg/ml) significantly reduced the serum levels of amylase and lipase (Fig. 2A and B).

### NJ reduced serum cytokine levels and pancreatic cytokine expression

**Figure 3:**
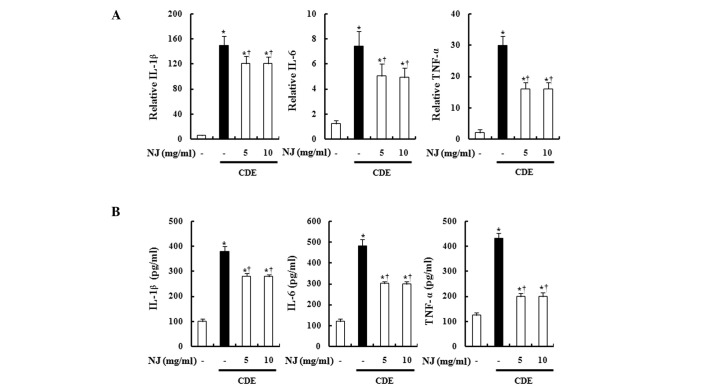
Effect of NJ on TNF-α, IL-1β and IL-6. To examine production of TNF-α, IL-1β and IL-6 (A) serum levels and (B) mRNA levels in the pancreas were measured by ELISA and real-time RT-PCR, respectively. Data are represented as the means ± SEM (n=6 in each group). ^*^P<0.05 vs. control group and ^†^P<0.05 vs. CDE-induced SAP mice. P<0.05 was considered to indicate a statistically significant result. The results were similar in 3 additional experiments. NJ, *Nardostachys jatamansi*; CDE, choline-deficient diet supplemented with ethionine; TNF, tumor necrosis factor; IL, interleukin; mRNA, messenger RNA; ELISA, enzyme-linked immunosorbent assay; RT-PCR, reverse-transcription polymerase chain reaction; SAP, severe acute pancreatitis.

It has been reported that ILs and TNFs increase during AP (7,8). Therefore, we examined cytokine levels in the serum and pancreas. The serum and pancreatic TNF-α, IL-1β and IL-6 levels were increased in mice with CDE-induced SAP. NJ treatment significantly reduced the levels of TNF-α, IL-1β, and IL-6 in the serum and pancreas (Fig. 3A and B).

### NJ inhibited activation of pancreatic MAPKs during CDE-induced SAP

**Figure 4:**
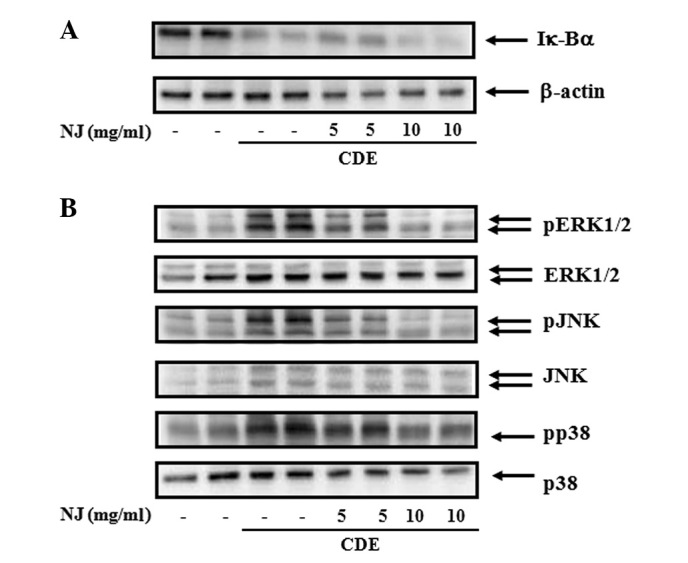
Effect of NJ on NF-κB and MAPK activation. Pancreata were obtained for western blotting to detect (A) Iκ-Bα and (B) MAPKs. Representative data from 1 of at least 3 separate experiments are shown. NJ, *Nardostachys jatamansi*; Iκ-Bα, inhibitory κ-Bα; MAPK, mitogen-activated protein kinase; CDE, choline-deficient diet supplemented with ethionine; (p)JNK, (phospho-)c-Jun N-terminal kinases; (p)ERK, (phospho-) extracellular signal-regulated kinases.

We assessed the effect of NJ on the activation of NF-κB (Fig. 4A) and MAPKs (Fig. 4B) in the pancreas. NF-κB activation was assessed by Iκ-Bα degradation (9). The CDE caused Iκ-Bα degradation, indicating an activation of NF-κB. NJ treatment did not inhibit Iκ-Bα degradation, suggesting that the anti-inflammatory effect of NJ was not associated with NF-κB (Fig. 4A). MAPKs were also activated during SAP. NJ treatment significantly reduced SAP-induced activation of MAPKs (Fig. 4B).

## Discussion

In the present study, we investigated the protective effects of NJ on diet-induced SAP. NJ inhibited the CDE-induced pancreatic damage markedly and reduced the digestive enzyme secretion and cytokine productions significantly. NJ treatment also reduced MAPK activation in a dose-dependent manner. Our results show that NJ may be a candidate for the treatment of SAP.

In Figs. 1 and 2, CDE caused significant pancreatic inflammation and hyper-stimulation of amylase and lipase. When this model was first introduced, it was considered that a CDE inhibited the biosynthesis of lecithins, a major membrane constituent, and caused a blockage of the exocytosis of zymogen granules, leading to intracellular enzyme accumulation, acinar cell injury and enzyme leakage into the interstitium/blood, resulting in auto-digestion of the pancreas by the activated enzymes (10). The hyper-stimulated enzymes would attack the acinar cells, then the injured cells secrete the pro-inflammatory cytokines (11). In this study, NJ treatment inhibited CDE-induced pancreatic damage and digestive enzymes (Figs. 1 and 2), which indicates that the action of NJ is mediated by the inhibition of digestive enzymes.

In the present study, NJ treatment inhibited the pancreatic and serum IL-1β, IL-6 and TNF-α levels (Fig. 3). The production of pro-inflammatory cytokines, including IL-1, IL-6 and TNF-α, has now been shown in the majority of animal models of pancreatitis (12–14). Also, the degree of cytokine elevation correlates well with the severity of organ inflammation and destruction. Investigations into the origin of these peptides have demonstrated that intra-pancreatic cytokine levels reach concentrations several-fold higher than corresponding systemic levels, thus providing evidence that IL-1, IL-6 and TNF-α are produced within the pancreas during acute pancreatitis (12). Therefore, the inhibition of pro-inflammatory cytokines is critical to reduce the severity of SAP.

NF-κB and MAPKs play key roles in regulating the cytokines involved in acute inflammatory pancreatic diseases (15,16). The abnormal activation of NF-κB and MAPKs may promote the transcription of pro-inflammatory factors, including TNF-α, IL-1β and IL-6. In an extracellular signaling loop, TNF-α, IL-1β and IL-6 also activate NF-κB and MAPKs, further promoting inflammatory reactions. In the present study, mice with SAP showed increased TNF-α, IL-1β and IL-6 release via NF-κB and MAPK activation. NJ treatment did not inhibit NF-κB activation, but did inhibit activation of MAPKs, consequently inhibiting cytokine release. These data suggest that the effective pathway of NJ is via MAPKs and not NF-κB (Fig. 4).

Our results indicate that NJ has protective effects on SAP by inhibiting MAPK pathways, thereby inhibiting TNF-α, IL-1β and IL-6 production. NJ treatment also reduced neutro-phil infiltration into the pancreas and reduced levels of serum enzymes. Our findings suggest that NJ may be a candidate for SAP treatment.
